# Without a pinch of salt: effect of low salinity on eggs and nauplii of the salmon louse (*Lepeophtheirus salmonis*)

**DOI:** 10.1007/s00436-023-07890-8

**Published:** 2023-06-02

**Authors:** Andreas Borchel, Erna Irene Heggland, Frank Nilsen

**Affiliations:** Department of Biological Sciences, University of Bergen, Bergen, Norway

**Keywords:** Aquaculture, Copepod, Crustacean, Embryo, Hatching delay, Hyposalinity, Invertebrate, Oocyte, Parasite

## Abstract

The salmon louse is an economically important parasite on Atlantic salmon and poses a major threat to aquaculture. Several treatment methods have lost their effect due to resistance development in the lice. A rather new method for combatting sea lice is freshwater treatment where the various life stages of lice are differently affected by this treatment. In this study, we analyzed the effect of freshwater on the egg strings. A 3-h treatment with freshwater had a detrimental effect on the egg strings. First, the water penetrated the string, widening it, then entering the eggs and enlarging them. Finally, the ordered structure of the egg strings collapsed, and no alive animals hatched. Shorter treatments had a lower effectivity, and treatments with brackish water also showed milder effects. The egg strings were found to have a protective effect against low salinities, as hatched nauplii died rapidly under conditions that embryos survived. We also found that embryos react to low salinity on a molecular level by changing gene expression of several genes, when incubated in brackish water. Additionally, the hatching of embryos treated with brackish water was delayed in comparison to seawater controls.

**Supplementary Information:**

The online version contains supplementary material available at 10.1007/s00436-023-07890-8.

## Introduction

The salmon louse (*Lepeophtheirus salmonis* Krøyer, 1837) is an ectoparasite and feeds on skin and blood from Atlantic salmon (*Salmo salar* Linnaeus, 1758) and other salmonids (Boxaspen 2006). The species presents a big challenge for the aquaculture industry as it generates high costs (Costello 2009) due to treatments, decreased fish welfare, and reduced fish growth. Additionally, regulations aiming to reduce the impact of the salmon louse in fish farms on wild salmon prevent further growth of this industry sector in Norway, at least in areas with a high louse prevalence (Vollset et al. 2018). Classical treatment methods include using various drugs such as azamethiphos, deltamethrin, emamectin benzoate, and, more recently, hydrogen peroxide (Jensen et al. 2020). However, the louse has shown great ability to adapt to these treatments and has developed resistance against most of the applied anti-salmon lice medicines (Helgesen et al. 2019). As medical treatment efficacy has decreased significantly during the last years, various non-medical treatment methods have been developed and are now used at a higher frequency than the classical medical tools. These non-medical treatments include a variety of methods, like lasers shooting the lice off the fish (Bui et al. 2020), thermal treatments (Moltumyr et al. 2021), or applying freshwater (FW). FW treatment is considered promising, as it appears to be one of the most environmentally friendly approaches, as the release of the water from the vessels into the sea is unlikely to have significant impacts on the environment in contrast to the release of some chemicals that might have adverse effects on non-target species, even when diluted (Frantzen et al. 2020).

FW is not considered detrimental for the euryhaline Atlantic salmon. FW treatments are also effective in treating amoebic gill disease; thereby, FW might be useful against two parasite species simultaneously (Powell et al. 2015). However, the actual effectiveness of FW treatments on the salmon louse is still unclear, and several partly contradictory findings have been published. There seems to be a consensus that the copepodids, i.e., lice in the infective stage, have a low tolerance for FW. After a short time in FW, they die, while their tolerance to brackish water (BW) of different salinities varies between different publications and lice strains (Bricknell et al. 2006; Sievers et al. 2019; Andrews and Horsberg 2019). Adult lice, which are probably the main target of FW treatments, seem to be rather robust against FW when attached to a host. Minimal survival times of 3 days, 7 days, or up to 21 days have been reported for attached lice in lab-setting (Wright et al. 2016). On the contrary, several reports found an effect of FW treatment on the lice numbers in an aquaculture setting. One study found a significant reduction in the total lice number after FW treatment with a reduction from 7 lice/fish to 1 louse/fish (Powell et al. 2015). Mechanical pumping and crowding of the fish only led to a decrease to 4 lice/fish, indicating an increased effect when handling is combined with FW. A survey of fish health professionals showed that this group of people on average assumes that a FW treatment can reduce lice numbers by more than 70–90% (Hjeltnes et al. 2018). These findings contradict the conclusion from a lab-based study stating that “a 3-h FW flush treatment of Atlantic salmon did not significantly affect the survival or development of *L. salmonis*” (Stone et al. 2002). One might assume that the treatments in the field combine several factors: FW on the one hand and mechanical handling like pumping and collision between hosts during crowding on the other hand, creating a synergy and resulting in increased lice loss.

Another possibility to administer FW treatment is by creating a FW layer on top of the seawater within a net pen. The overall effect of this method is unclear, as fish seem to spend a too short time in this upper layer (Wright et al. 2018). FW treatment could target not only the adult lice themselves but also the egg strings (ESs) adult females are carrying. Salmon lice females carry several hundred eggs in ESs, attached to their body until the eggs hatch, after roughly 6.3 days at 12 °C (Hamre et al. 2019). Shortly after hatching, the female lice extrude a new ES pair and maintain ES production for its remaining lifespan. ESs do not hatch in FW, and hatching in BW up to 15 ppt prevents the development of active nauplii and copepodids (Johnson and Albright 1991). However, it has not been reported whether short-term treatments with FW or BW may influence ES hatching success. A FW layer on top of the net pens could lead to several frequent FW exposures for the ESs. A reduction in ES hatching might be beneficial for salmon aquaculture by reducing lice larvae production. Therefore, this study aimed to analyze how FW and BW influence the salmon louse egg hatching and the early life stages.

## Methods

### Animals

We used salmon lice of the laboratory strain LsGulen, which has been described before (Hamre et al. 2009). Lice were cultivated at the LiceLab facility at the University of Bergen. Atlantic salmon were kept in accordance with Norwegian animal experiment legislation and were infested with copepodids according to standard protocols. Adult female lice were harvested, and ESs were collected and placed in flow-through hatching wells as described before (Hamre et al. 2009). The water temperature was ca 9 °C.

### Experiments

#### Effects of low salinity on hatching

To test the effects of a short-term exposure to low salinity on hatching, we incubated ESs in different salinities for different durations. The experiments were performed with BW (7.5 ppt) (*n*=6) and FW (*n*=5). We cut the ESs into three parts of similar size with a scalpel and afterwards took photos to determine the number of eggs in each ES piece. The pieces were then transferred to the desired salinity. Incubation times were 5 h, 3 h, or 0 h (control). The 0-h controls were exposed to stagnant SW for 5 h. The 3-h samples were exposed to stagnant BW or FW for 3 h, followed by 2 h in stagnant SW, while the 5-h ESs were exposed to 5-h stagnant BW or FW. After 5 h, all samples were transferred from stagnant water back to running SW. After 10 days, new photos from the contents of the hatching well were taken and the number of nauplii and copepodids determined. For the analysis, we calculated the ratio of hatched eggs (total number of animals/number of eggs) and the ratio of animals molted to the copepodid stage (number of copepodids/number of eggs).

Mann-Whitney Rank Sum tests were executed to determine statistical differences between 3- and 0-h incubation (control), as well as 5-h and 0-h incubation.

The same type of experiment was performed with a shorter incubation (1h, FW, *n*=7) and lower salinities (4h, 6–7 ppt and 3–4 ppt, *n*=16). Here, the ESs were observed for hatching directly in the hatching wells, without accurate counting of the animals.

#### Visible effects of FW on ES integrity

To evaluate the effect on an ES upon FW exposure, whole ES pairs (*n*=3) were used and photographed under a dissecting microscope (Axio Zoom.V16, Zeiss, Oberkochen, Germany). From each ES pair, one string was transferred into FW, while the other remained in SW. After 1, 3, and 5 h, new photos of the ES were taken in FW or SW, respectively. ES diameter and egg thickness were measured using the Zen 3.1 software (Zeiss). ES diameter was determined based on measurements of two eggs on two photos of different positions each. Egg thickness was determined by measuring the distance from the outer boundaries of five eggs on two pictures and dividing this distance by five. The positions for the photos were randomly selected within the middle of the ESs. For the statistical analysis, paired *t*-tests were carried out to detect differences between SW- and FW-incubated ESs after the different incubation durations, for both ES diameter and egg thickness.

#### Hyposalinity-induced hatching delays

To better understand the hatching delays observed in several incubations, we varied the incubation times and salinities during incubation. Several (23) ESs were cut into two or three parts and then each part was transferred into a different salinity. One part of each ES was incubated in SW, the other parts either in 17 ppt, 21 ppt, or 25.5 ppt. Incubation time was either 24, 48, or 72 h, before the ES parts were transferred into incubators with continuous SW flow. Hatching of the ES parts was then monitored at least once a day, usually more frequent, until all ES parts had hatched. For the analysis, we counted the number of observations in which BW-treated ES pieces hatched earlier or later than their corresponding SW controls. In this analysis, data from additional experiments with only 4-h incubation at 17 ppt and 8.5 ppt were included, for which the time of hatching had also been observed. In total, the hatching of 65 BW-treated ES parts was observed and compared to their respective controls. Additionally, we quantified the observed hatching delay. To calculate the minimum hatching delay for each treatment, we subtracted the time at which hatched animals were found in the SW control from the last time point at which no hatched animal was observed within the BW-treated group. The maximum hatching delay was calculated by subtracting the last observation time at which no hatched animals were observed in the SW control from the time point of first observation of hatched animals in the BW-treated group. Only ESs for which we could pinpoint the hatching delay with an observational uncertainty of maximum 18 h are reported, yielding the following number of observations per treatment: 17 ppt, 48 h, *n*=6; 17 ppt, 24 h, *n*=4; 21 ppt, 72 h, *n*= 3; 21 ppt, 48 h, *n*=3; 25.5 ppt, 24 h, *n*=4. When ESs were observed during the hatching process, we assumed that hatching had started maximum 4 h before, unless we had an observation of an intact ES from less than 4 h before. Statistical significance was analyzed by one-sample *t*-test comparing the observed minimum and maximum hatching delay with an expectation of 0 h hatching delay, which would be expected without an effect of the treatment. Additionally, *t*-tests were performed to check for differences between incubation duration within one salinity, and between salinities within the same incubation duration.

#### Molecular reactions of BW-treated ESs

In the next experiment, we wanted to test if eggs react to a low-salinity exposure on a molecular level. ES pairs (*n*=9) were cut in half, and two halves were incubated for 24 h in BW (12 ppt), while the others were incubated in full SW. One ES half of each condition was then removed from the water, blotted dry on paper, and frozen at −80 °C, whereas the other halves were transferred back to full SW and controlled for hatching. To isolate RNA from the frozen ESs, these were transferred to 1 ml TRI reagent and homogenized with ceramic beads (1.4 mm) in a TissueLyser II bead mill (Qiagen), with a frequency of 30 Hz for 3 min. After adding 200 μl chloroform and subsequent phase separation, the aqueous phase was used as input material for the Zymo Direct-zol RNA Microprep kit, following the manufacturer’s instructions and including an on-column Dnase treatment. Three hundred nanograms of RNA (as determined by Nanodrop spectrophotometric measurement) was employed in cDNA synthesis using a mixture of Oligo(dT) and random hexamer primers and the AffinityScript QPCR cDNA Synthesis Kit in 10-μl reactions. The resulting cDNA was diluted 1:10, and 2 μl was used for 10-μl qPCR reactions (equaling 6 ng RNA equivalents per reaction) employing PowerUp SYBR Green Master Mix (Thermo Fisher Scientific). Primer concentration was 500 nM for forward and reverse primer, respectively. We used elongation factor 1 alpha as well as RPS13 as reference genes, as they had been shown to be unaffected by low salinities in adult salmon lice before (Borchel et al. 2021). Thermocycling was performed on QuantStudio 3 Real-time PCR machines (Applied Biosystems), with the program: initiation, 50 °C, 2 min; holding, 95 °C, 2 min; 40 cycles of 95 °C, 15 s and 60 °C, 1 min, concluded by a melting curve. As targets, we analyzed a selection of eight genes that had been found to be regulated upon exposure to low salinity in adult female salmon lice before (Borchel et al. 2021), given in supplementary file 1, together with the used primer sequences. All genes are identified by their Ensembl stable ids (EMLSAG…) based on the salmon louse genome LsalAtl2s (Skern-Mauritzen et al. 2021). Calculations of the relative gene expression took into account the PCR efficiencies of the primers, and were relative to the expression of the reference genes EF1A and RPS13. All expression values were normalized with the lowest median expression for each gene.

In a second experiment, BW (17 ppt, 24 h)-treated ESs (*n*=3) were allowed to hatch and the nauplii were collected in RNAlater and analyzed by qPCR as described above. Collection of the nauplii took place within 24 h after hatching.

Significant differences between BW and SW controls were identified using paired Wilcoxon signed-rank tests.

#### Low-salinity effects on nauplii

To analyze the effects of low salinity on nauplii, we checked ESs several times a day for hatching. The ESs were incubated in flow-through hatching wells with SW at ca. 9 °C. A small number (10–20) of nauplius I animals were transferred with a pipette into the removed lid of a 1.5-ml reaction tube. The liquid was then removed carefully with a smaller pipette, preventing the uptake of lice. When the animals lay dry, 150 μl FW was added as quickly as possible. Animals were observed under a stereo microscope, videos were recorded, and photos taken with an attached digital camera. The experiment was repeated with animals that had already reached the nauplius II stage. These animals were exposed to different salinities (0, 7.5, 15, 22.5, 34 ppt) and photographed every 30 s for 15 min (*n*=3). Afterwards, we counted the number of leaking animals in each photo and calculated their fraction of all animals. To validate our findings and investigate the effects of a very short low-salinity exposure on the earliest life stages, we divided Nauplius II larvae from the same ES into different hatching wells. These hatching wells were then transferred into water of different salinities (0, 7.5, 15, 22.5, 34 ppt) for 10 min, before being transferred back into SW and flow-through of the water was reestablished. The hatching wells were then regularly checked for molting of the animals to the copepodid stage (*n*=6). The obtained data was fitted by nonlinear regression to a sigmoidal, logistic four-parametric function.

### Statistics

Mann-Whitney Rank Sum tests, the non-linear regression, and *t*-tests were executed in Systat Sigmaplot 14. The gene expression data were analyzed for statistical differences between BW treatment and control using the “wilcox.test” function, from the “ggpubr” package (Kassambara 2020) for R (R Core Team 2020), performing paired Wilcoxon signed-rank tests.

For all statistical tests, *p*-values below 0.05 were considered statistically significant.

## Results

### Effects of low salinity on ESs

**Fig. 1:**
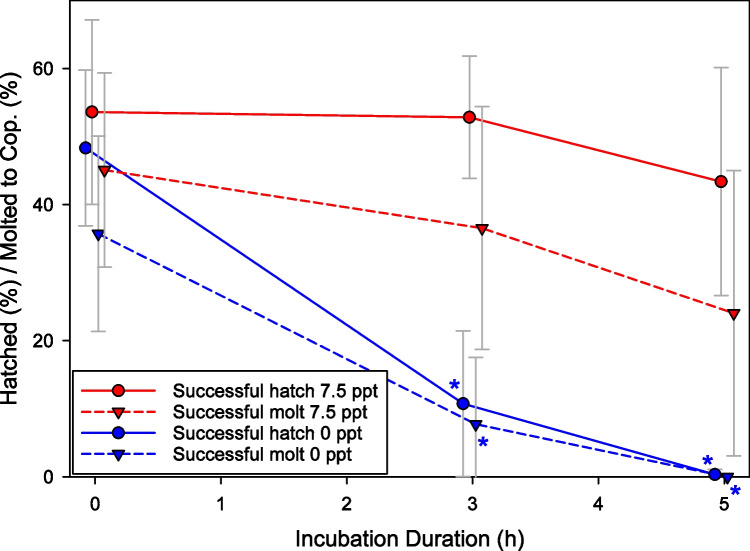
Hatching and molting of FW- or BW-treated ESs. ESs were cut in three parts which were incubated in FW (*n*=5) or BW (7.5 ppt) (*n*=6) for 0, 3, or 5 h. Circles with solid lines show the average rate of hatched animals, triangles with dashed lines the rate of copepodids at the time of sampling. Asterisks mark a statistically significant difference (*p*≤0.05) of the marked value in comparison to the respective control (0-h incubation; Mann-Whitney Rank Sum Test). Error bars give standard deviation. Values are shifted on the *x*-axis around the actual incubation duration for better visibility of the data

A FW incubation of ESs for 5 h completely prevented hatching, while an isochronal incubation in BW of 7.5 ppt did not have any significant effect on hatching or molting (Fig. 1). FW on the contrary had already a significant effect after 3 h with a reduction of hatched animals by 78% and of molted copepodids by also 78%.

In another experiment, we tested the effect of 1-h incubation in FW on ES hatching. Only one out of seven ESs was affected and this ES was very dark and immediately before hatching; for the other six ESs, hatching success comparable to SW controls was observed. To enhance comprehension of the onset of molting issues in ESs at reduced salinity levels, we investigated two supplementary salinity levels (6–7 ppt; 3–4 ppt). Incubating ESs in a salinity of 6–7 ppt for 4h, 11 out of 16 ES parts (69%) hatched comparable to the SW control, for which many animals were found swimming in the hatching well and only neglectable numbers were seen stuck within the ES remains. In one case, there was not a single animal that had hatched, and in the last four cases, only few animals hatched, several of them severely malformed. Lowering the salinity even more (3–4 ppt), effects on hatching became more obvious. Five out of 16 cut ESs (31%) did not hatch at all and did not produce any viable offspring. For the other ESs, only few eggs (less than 30%) hatched, several eggs not hatching could be observed within the ES, and several animals were floating around within the egg membranes. The ES pieces incubated in SW hatched to a high degree, with many animals swimming actively in the hatching wells. In every treatment group with successful hatching, animals molted into the copepodid stage.

### Visible effects of FW on ES integrity

**Fig. 2:**
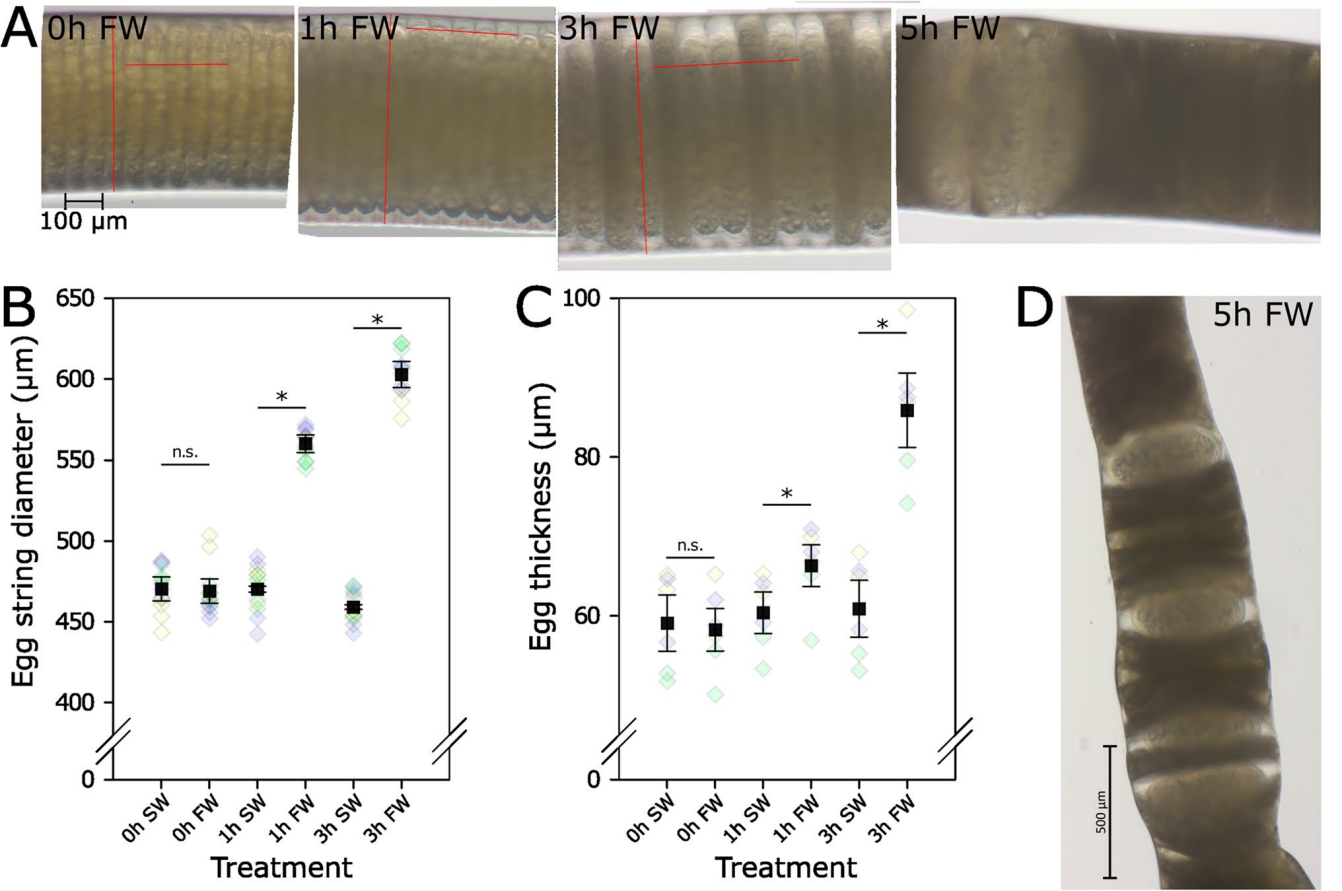
Effect of FW on ESs of *L. salmonis*. **A** Micrographs of ES after FW treatment for 0–5 h. Red lines mark the egg string diameter and the thickness of five eggs used to calculate egg thickness. All pictures in (A) were taken with the same magnification. **B** and **C** ES diameter **B** and egg thickness **C** after 0–3 h FW and SW treatment. The black squares give the average ± SEM (*n*=3); the diamonds in the background give the values of several measurements (4 measurements per ES for diameter, 2 for thickness). Diamonds with the same color come from the same pair of ESs. Asterisks (*) mark significant (*p*≤0.05) differences (paired *t*-test). **D** A lower scale photo of another ES after 5 h FW treatment showing swelling of the ES and alteration of the egg shape

Examinations of FW-treated ESs under a stereomicroscope revealed that the diameter of the ES increased during the FW incubation period, while there was no change in the diameter of the corresponding ESs incubated in SW (Fig. 2). Within the first hour, there was a strong increase in ES diameter (19%). The relative increase within the next 2 h was lower (an additional 8%, comparing diameter at 3 to 1 h). After 3 h, differences in the shape of some eggs became apparent. Most eggs were surrounded by a space between egg membrane and ES, while other eggs, which appeared thinner, lacked this space and stretched out fully to the borders of the ES. Egg thickness was also affected by FW treatment. The increase in thickness was low (14%) within the first hour and higher (an additional 29%) at the next analyzed time point. Assuming a cylindrical shape of the eggs, one can calculate that the average egg volume after 3 h increases by a factor of 2.4.

### Hyposalinity-induced hatching delays

**Fig. 3:**
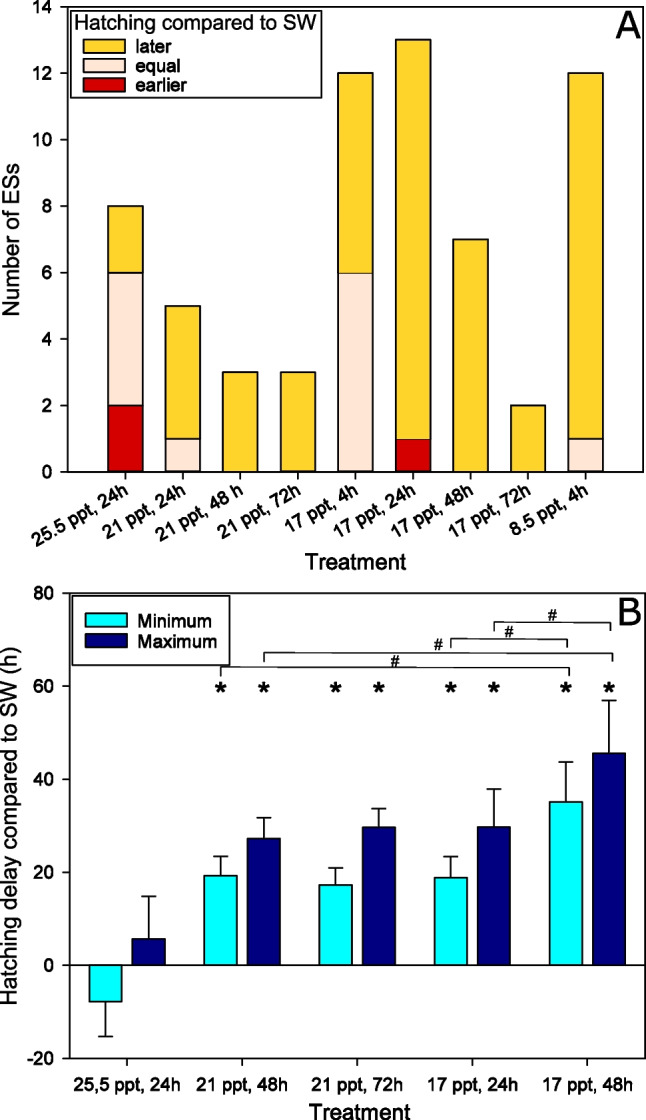
Hatching delay caused by low salinities. ESs were cut in 2 or 3 parts which were exposed to SW or BW of different salinities for different durations. **A** For every condition, the bar represents the total number of ESs, subdivided into the number of ESs, for which the BW-exposed ES part was found hatched, but the SW control was still intact (earlier), both ES parts had hatched (equal) or only the SW control had hatched at the time of observation (later). **B** Based on frequent surveillance of hatching, the minimum and maximum hatching delay of BW-treated ES pieces in comparison to their SW counterparts was determined (*n*=3–6). A hatching delay of 0 h indicates that SW- and BW-treated ES part hatched at the same time point, a positive hatching delay indicates that SW-treated ES parts hatched first, and a negative value that BW-treated ES parts hatched first. Asterisks mark minimum or maximum hatching delays significantly (*p*≤0.05) different from 0 h (one sample *t*-test); the brackets with hashtags mark significantly (*p*≤0.05) different delays between the two marked treatments (*t*-test).

BW-incubations of ESs also had an influence on the timing of hatching (Fig. 3). Incubations in water of 21 ppt and lower for 1 day led to a later hatching of BW-treated ES parts compared to SW controls. In 17 ppt and below, we observed this also after incubations of just 4 h (Fig. 3A). Only the group that was incubated in 25.5 ppt for 24 h did not show a shift towards delayed hatching but earlier, equal and later hatching were observed with similar frequencies. We found statistically significant hatching delay durations for treatments with 21 ppt and below, but not for 25.5 ppt (Fig. 3B). A 48-h incubation in 17 ppt gave a statistically significant longer hatching delay in comparison to 21 ppt. At 17 ppt, a longer incubation time (48 h vs 24 h) also delayed hatching significantly longer.

### Molecular reactions of BW-treated ESs

**Fig. 4:**
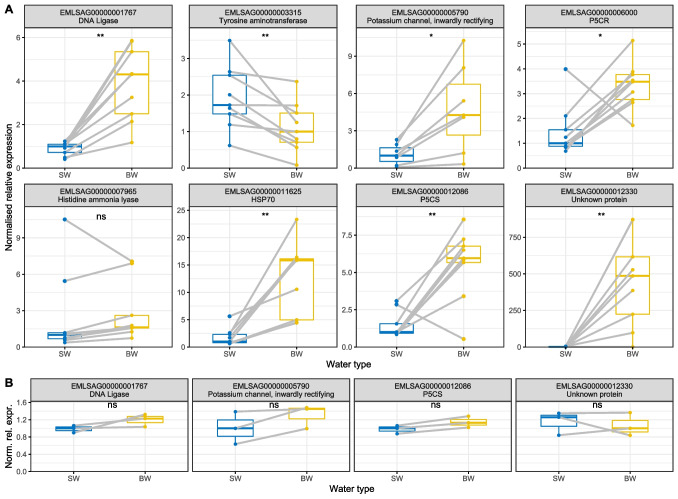
Gene expression upon BW treatment. Egg strings were cut in half and one-half of each ES was incubated in regular SW or BW for 24h. Gene expression of genes known to react to changed salinities in adult lice was measured. **A** Gene expression levels in ESs (*n*=9) directly after 24 h BW (12 ppt) exposure. **B** Gene expression levels in nauplii larvae samples (*n*=3), which have been exposed to BW (17 ppt) for 24 h as embryos inside their ES, within 24 h after hatching. Significance levels (paired Wilcoxon signed-rank test): *p≤0.05; **p≤0.01; ns *p*>0.05. For EMLSAG00000005790, two values are not displayed, as corresponding mRNA was only detected in BW-treated but not in SW-treated egg strings. The lines link the expression values of the two halves of each individual ES

On the molecular side, we found changes in gene expression within ESs upon a 24-h BW treatment (Fig. 4A) in comparison to SW. In BW (12 ppt), gene EMLSAG00000001767, encoding a DNA ligase, was upregulated on average 4-fold, and EMLSAG00000011625, encoding HSP70, was upregulated ninefold. In addition, two genes involved in proline-synthesis showed an upregulation of fivefold (EMLSAG00000012086, Delta l-pyrroline-5-carboxylate synthetase) and threefold (EMLSAG00000006000, Pyrroline-5-carboxylate reductase), respectively. A potassium channel (EMLSAG00000005790) was upregulated 4.7-fold and a protein of unknown function, formerly found to be upregulated in low salinity in adult salmon lice (Borchel et al. 2021), was upregulated very strongly (EMLSAG00000012330; 317-fold). The trend of upregulation of a histidine ammonia lyase (EMLSAG00000007965) was not statistically significant and a tyrosine aminotransferase was slightly downregulated (EMLSAG00000003315, 0.6-fold).

To test if the identified gene expression differences between BW-treated and SW-treated ES outlast the transfer back to SW and subsequent hatching, we measured the gene expression of four genes in nauplius I larvae that hatched from BW-treated ESs. While we observed a hatching delay of roughly 1 day for the BW-treated ESs, the expression of the four genes was equal between BW-treated animals and their respective controls (Fig. 4B).

### Low-salinity effects on nauplii

**Fig. 5:**
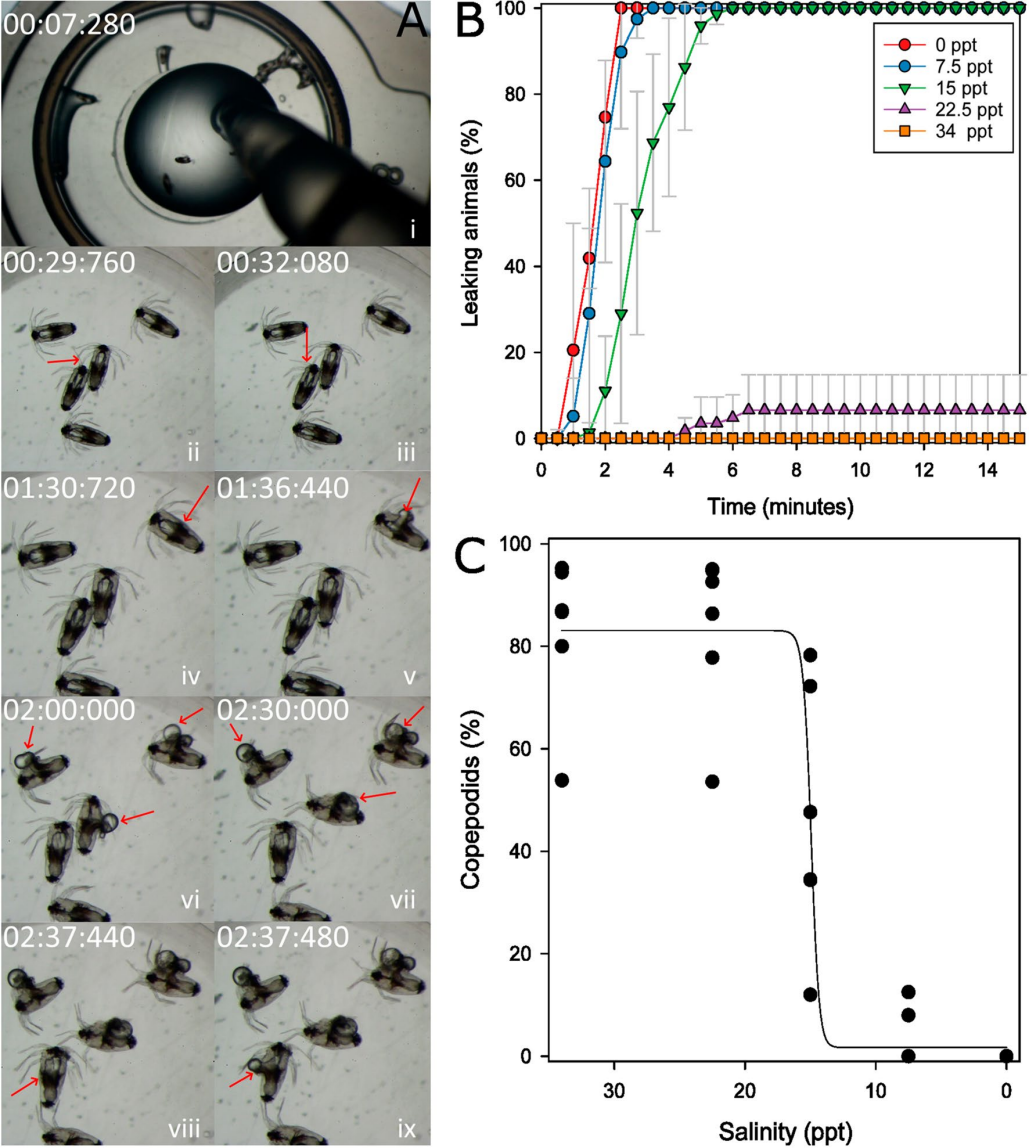
**A** Reaction of *L. salmonis* nauplius I larvae to FW. Still images from a video recorded during exposure (Supplementary File 2); time stamps are given for each image. Red arrows mark the regions described in this figure text. (i) After transferring the animals to a reaction tube lid, the SW was removed and replaced by FW. (ii–iii) After 30 s, animals are barely moving, but some antenna movement is still visible. (iv–v) The first animal rips open after roughly one and a half minutes. (vi–vii) More animals rip open, and more fat keep leaking out from the first affected animals. (viii–ix) The opening happens explosion-like, where a big sphere of spilled fat leaves the animal’s hitherto intact body within 20 ms. **B** Leaking of nauplius II larvae in different salinities. Nauplius II larvae from different ES pairs (*n*=3) were exposed to different salinities and photos were taken every 30s for 15 min. The number of leaking animals was determined on each photo. Values are means ± SD. **C** Molting capacity after a 10-min incubation in different salinities. Nauplius II larvae from several ESs (*n*=6) were incubated in different salinities for 10 min and then transferred back into running seawater. After several days, nauplii and copepodids were counted. Every dot represents one experiment. The line shows a logistic regression (*R*^2^=0.8732)

We also tested the effect of low salinities on *L. salmonis* nauplius I and nauplius II larvae. The effects of FW were rapid and strong (Fig. 5A). In less than 3 min after the addition of FW to nauplius I larvae, the shells of the animals ripped open, and yolk and fat were running out of the animals. We observed such leakage of inner body fluids also in BW with salinities up to 17 ppt; in water with 25 ppt, the animal remained intact. Nauplius II larvae exposed to BW below or equal 15 ppt showed similar leaking of body fluid as nauplius I larvae (Fig. 5B). The rate and intensity of the body fluid leakage were inversely related to the salinity, with low salinities displaying the strongest and quickest effects. The animals exposed to 15 ppt ripped open ca. 1 min later than animals exposed to 7.5 or 0 ppt. Very few animals (<10%) at 22.5 ppt ripped open, and the SW controls all remained intact during the 15-min-long observation period. Even though the majority of animals exposed to 22.5 ppt remained intact, the recorded photos revealed that they suspended any movement during the incubation time, in contrast to the SW controls, which were actively swimming.

A 10-min incubation in BW had effects on the molting capacity of nauplius II larvae (Fig. 5C). Animals incubated in 22.5 ppt BW molted at the same rate as SW controls, while incubation at 7.5 ppt or below led to the almost complete absence of copepodids. An incubation at 15 ppt prevented approximately 50% of the nauplii to molt to the next life stage.

## Discussion

### The egg string protects the eggs from the environment

Our results show that embryos within an ES are less sensitive to low salinities than hatched nauplius larvae indicating that the ES has a protective function. Incubation of an ES in FW for 1 h did not influence the hatching of the eggs, whereas a FW treatment of the hatched nauplius I or II larvae for only 5 min resulted in breaking of the shell and release of internal body content. An ES incubated for 24 h at 12 ppt BW hatched to some degree, whereas nauplii ripped apart after less than 15-min exposure to BW of 17 ppt. Overall, our results point to the nauplii stages as the most sensitive stages for low salinity, as suggested before (Gravil 1996). Fish-attached adults can survive in FW for at least a week (Hahnenkamp and Fyhn 1985); copepodids are far more susceptible to reduced salinity. A recent study found EC_50_ values between 10 and 18 ppt for copepodids after 24-h exposure and between 2 and 4 ppt for pre-adult II lice (Andrews and Horsberg 2020). Another study found free-swimming copepodids to be somewhat more sensitive to low salinity, where an LT_50_ value of 4.5 h was determined for water with 16 ppt. For FW (1 ppt), the LT_50_ value was 0.4 h (Sievers et al. 2019). Host-attached copepodids appear to be more robust (Sievers et al. 2019). Montory et al. (2018) found an interaction between salinity and temperature in survival for *C. rogercresseyi* and this could also be the case in *L. salmonis*. The measured tolerances of copepodids vary between studies, but they are higher than the tolerance we observed for nauplii. Although copepodids also die in FW in a short time, the addition of just some SW is enough to increase the survival time to hours (e.g., a salinity of 9 ppt gives a LT_50_ of 2h; (Sievers et al. 2019)), whereas nauplii rip open within minutes. One possible explanation for this might be differences in the cuticle of nauplii and copepodids. The cuticle of the copepodids might be more solid than the cuticle of the previous naupliar stages. The explosion-like opening of the nauplii at low-salinity water suggests that this is due to physical force and that the cuticle has not evolved to resist such osmotic pressure created by low-salinity water. A robust cuticle in copepodids might prevent this strong and rapid physical damage, but ion loss and inability to regulate cell size will eventually result in death. An additional explanation might be different osmolarity of nauplii and copepodids. As the larval stages are lecithotrophic, maternal resources are consumed during the free-living stages. This might lead to a reduced osmotic difference between in- and outside for copepodids reducing the osmotic forces. The difference in tolerance towards BW and FW can also cause behavioral differences between nauplius larvae and copepodids. In an experiment where *L. salmonis* larvae could choose to remain in a SW layer or to cross through a halocline into BW with different salinities, nauplii almost completely avoided water below 30 ppt, whereas some copepodids voluntarily swam into BW with as low as 16 ppt (Crosbie et al. 2019). According to our data, this makes sense, as nauplii would die quickly under these salinities. Copepodids are more resistant to reduced salinity and are able to attach to a host at 26 ppt (with 45% efficiency of SW) or even 19 ppt (34%) (Bricknell et al. 2006).

The ES and the egg membrane seem to be a somewhat efficient barrier that at least reduce the effects of the osmotic pressure on the eggs. Nevertheless, our ES measurements show that FW enters the ES, first increasing its diameter and then the egg thickness. The observed gap between egg and ES wall might serve as a buffer, preventing the direct entry of FW into the eggs. The observed egg thickness for the untreated ESs was in the size range of eggs obtained from the field (Gravil 1996). How the ES performs its protective function is unclear. Some components of ESs have been described (Borchel et al. 2019), but the structure has not been resolved so far. A previous study (Gravil 1996) also observed the swelling of ESs in FW and they reported that ESs exposed to FW burst 4 min after exposure. We only observed such bursting when treating very dark ESs that were close to hatching. The timeline of the microscopic observations aligns well with the hatching success observed for FW-treated ESs. After 3 h, the hatching success dropped and we observed differently shaped eggs within the ES. This suggests that the effects of low salinity on whole and cut ESs are similar.

When it comes to FW treatment in the field, the protective effect of ESs might likewise be too weak for embryo survival. When FW treatment is performed onboard a well-boat, treatment duration is usually between 5 and 8h (Groner et al. 2019) and a stronger effect on hatching could be anticipated due to the increased exposure time. Even if a proportion of the adult females survive the treatment, our results show that the ESs will be destroyed, and larval production reduced.

### *L. salmonis* embryos react to their environment

To our knowledge, changes in gene expression in response to environmental stimuli in *L. salmonis* have only been demonstrated in nauplius II larvae and older animals. Harðardóttir et al. (2019) showed a slight downregulation of a chitin synthase upon treatment of nauplius II larvae with chitin synthesis inhibitors. Strong induction of heat shock proteins was found upon heat shock, and the nauplii also harden after a sub-lethal temperature increase, leading to a higher thermotolerance (Borchel et al. 2018). In this study, we identified a gene expression response to an environmental stimulus present already in the earliest life stage of the salmon louse, i.e., the embryo within the egg string. Versatile developmental gene expression changes during embryogenesis have been described in various species like the mummichog (Bozinovic et al. 2011) or the fruit fly (Tomancak et al. 2002). For mammalian embryos, adaptive plasticity has been shown, consisting of reactions to the environment at several physiological levels (Thompson et al. 2006). For example, mouse embryos react in vitro to reduced oxygen levels in their incubator by increasing the gene expression of glucose transporters (Kind et al. 2005). Also, bovine embryos showed a regulation of gene expression under oxidative stress (Amin et al. 2014). Embryos of the fish species *Epinephelus moara* modify gene transcription in response to cold temperature (Chen et al. 2020). Reports on embryo plasticity in invertebrates are less common. One exception is the sea urchin *Strongylocentrotus purpuratus*. In this organism, gene expression changes in different embryonal stages have been found depending on CO_2_ level (Hammond and Hofmann 2012) and temperature (Runcie et al. 2012). In a coral species, the temperature had an effect on the embryo gene expression (Voolstra et al. 2009), and cadmium exposure of annelid embryos influenced their gene expression (Gomes et al. 2018). However, to our knowledge, this study is the first to show embryonic cellular reactions to a reduced salinity and additionally the first to describe embryonic plasticity on gene expression level in copepods. The genes upregulated in low salinity were chosen based on their salinity-dependent regulation in adult lice (Borchel et al. 2021). The finding that most of these genes are upregulated in embryos as well suggests that these genes belong to a stage-independent hyposalinity response of the salmon louse and it is possible that these genes are responsive in all life stages. At the same time, the downregulation of a tyrosine aminotransferase (which is upregulated in BW in adult salmon lice (Borchel et al. 2021)) shows that the various life stages react slightly different towards a change in salinity.

The observed upregulation of several genes after BW treatment of ESs was only measurable in the ESs, directly upon treatment, whereas no upregulation was detected in the nauplii hatched from BW-treated ESs. This suggests that the upregulation of the analyzed genes is a direct, temporary response to low salinity, which is diminishing following return to regular salinity conditions.

The exact functions of the analyzed genes in osmoregulation and survival at low salinities are not known yet. Among the upregulated genes was a HSP70-encoding gene. Specifically, this gene has been found to be strongly induced by heat shock, but also by decreased salinity in copepodids before (Sutherland et al. 2012 [25/26 ppt, 24h]; Borchel et al. 2018 [9 ppt, 20 h]). An induction of HSP70 suggests an activation of the cellular stress response, which might indicate damages in cellular structures and the need for protein refolding. The DNA ligase we found upregulated in BW-treated ESs might have a similar function. DNA ligase III has been found to protect cells against oxidative stress (Akbari et al. 2014). Apart from one gene encoding an unknown, signal-peptide-carrying protein with no strong homology to proteins of known function, which was extremely upregulated after BW treatment, four other analyzed genes encode enzymes involved in amino acid metabolism. Two of them, P5CR and P5CS, form the proline biosynthetic pathway which uses glutamate as a substrate to form proline. An upregulation of these two enzymes suggests an increase in the capacity to synthesize proline, which is among other things known as an osmolyte in plants (El Moukhtari et al. 2020). Apart from the salmon louse (Borchel et al. 2021), another copepod, *Tigriopus californicus*, has been found to upregulate these two genes in hypoosmotic environments (Lee et al. 2021), suggesting a common function. The other two analyzed enzymes (tyrosine aminotransferase and histidine ammonia lyase) are part of two different pathways which produce glutamate that might be used as substrate for proline synthesis. However, one of the enzymes was statistically unaffected by hyposalinity, while the other was downregulated, suggesting that their regulation is independent of proline biosynthesis. The last analyzed gene encodes for an inwardly rectifying potassium channel and was found to be upregulated in BW. Such channels allow for the transfer of K^+^ ions into the cells (Chen and Swale 2018). Therefore, they might be important to maintain the potassium-concentration within salmon louse cells under hyposaline conditions.

In addition to the allegedly specific responses to a low salinity by expressing a specific set of genes, we observed an additional effect, the delay of hatching by a FW or BW treatment of ESs. A 4-h incubation in FW led to a hatching delay of several hours and when incubating the ESs in BW for a day, the hatching delay increased roughly to the same time span. It is known that the hatching rate of salmon lice is influenced by temperature, with earlier hatching in higher temperature (Boxaspen and Næss 2000; Hamre et al. 2019). This temperature dependency can be easily explained by the temperature dependence of enzymes and metabolism, while a direct relation to salinity is less apparent. However, for the copepod *Eurytemora affinis*, a relation between developmental time of hatched animals and salinity has been shown (Karlsson et al. 2018) as well. How the developmental slow-down in salmon lice is mediated and why it occurs remains unknown. It might be beneficial for the embryos to slow down development and hatching until they are in more favorable conditions, as hatching in too low salinity might kill the hatching animals. Hence, a reduced developmental speed (i.e., longer time until hatching) gives longer time for the female on the host to be transported back into seawater. Our results showed that a salinity of 25.5 ppt did not influence the hatching timing, whereas a salinity of 21 ppt or lower did. Within this range, nauplii have their tolerance limit for reduced salinity. While successful hatching was observed at both 20 and 25 ppt, a higher ratio of dead animals was observed in 20 ppt and a significantly higher number of animals reached the nauplius II stage in 25 ppt compared to 20 ppt (Gravil 1996). Therefore, it might be beneficial to delay hatching under circumstances that are detrimental to the hatched animals. Additionally, the duration of the exposure plays a role for the extent of the hatching delay, at least at 17 ppt. There was not a direct 1:1 relationship between incubation time and the duration of the hatching delay. For example, incubation at 21 ppt for 2 or 3 days led to delays of roughly 1 day, only. Overall, this suggests that development in BW is not completely halted but rather slowed down by low salinity. This is a major difference from the diapause eggs that are produced by many copepods under detrimental environmental conditions (Holm et al. 2018) that can last decades or centuries before hatching (Hansen 2019). It falls more in the category of quiescent eggs, which are “related to fast and unpredictable changes in living conditions” (Holm et al. 2018), even though the quiescence effect seems to be rather limited in salmon lice, where the development is not completely halted. This observation is especially interesting as in the sea louse species *C. rogercresseyi *no effect of salinity on hatching time was observed (Montory et al. 2018), which might suggest a specificity of the hatching delay for the salmon louse. However, in the *C. rogercresseyi* study, ESs were exposed permanently to different salinities. We cannot exclude that the observed hatching delay is caused by the return of the ESs from low to high salinity instead of the low salinity itself.

## Conclusions

In this study, we showed that the egg strings of salmon lice are resisting freshwater treatment for a short period of time. A freshwater treatment duration of 5 h was reliably destroying egg strings. However, when considering freshwater as a treatment method, the technical procedures must ensure that the treatment freshwater is only contaminated with spurious amounts of seawater, as already relatively small salt concentrations during the incubations had a positive effect on hatching success. We demonstrated that embryos react to a reduction in salinity by upregulating several genes that might help them adapt to low salinity conditions. At the same time, hatching of brackish water–treated egg strings is delayed. Nauplii are the most susceptible life stage, being far more sensitive to reduced salinities than both egg strings and copepodids. Exposure of nauplii to low salinities for only a few minutes might be very effective in the prevention of salmon lice infestations.

## Supplementary information

ESM 1 Supplementary File 1: Genes analyzed, primers used and their efficiency.

ESM 2 Supplementary File 2: Video of freshwater exposed nauplius I larvae. See Figure 3.

## Data Availability

The authors confirm that the data supporting the findings of this study are available within the article and its supplementary materials.

## References

[CR1] Akbari M, Keijzers G, Maynard S (2014). Overexpression of DNA ligase III in mitochondria protects cells against oxidative stress and improves mitochondrial DNA base excision repair. DNA Repair (Amst).

[CR2] Amin A, Gad A, Salilew-Wondim D (2014). Bovine embryo survival under oxidative-stress conditions is associated with activity of the NRF2-mediated oxidative-stress-response pathway. Mol Reprod Dev.

[CR3] Andrews M, Horsberg TE (2019). Sensitivity towards low salinity determined by bioassay in the salmon louse, Lepeophtheirus salmonis (Copepoda: Caligidae). Aquaculture.

[CR4] Andrews M, Horsberg TE (2020) Sensitivity towards low salinity determined by bioassay in the salmon louse, *Lepeophtheirus salmonis* (Copepoda: Caligidae). Aquaculture. 10.1016/j.aquaculture.2019.734511

[CR5] Borchel A, Heggland EI, Nilsen F (2021). The transcriptomic response of adult salmon lice (Lepeophtheirus salmonis) to reduced salinity. Comp Biochem Physiol - Part D Genomics Proteomics.

[CR6] Borchel A, Komisarczuk AZ, Rebl A (2018). Systematic identification and characterization of stress-inducible heat shock proteins (HSPs) in the salmon louse (*Lepeophtheirus salmonis*). Cell Stress Chaperones.

[CR7] Borchel A, Kongshaug H, Nilsen F (2019). Identification and description of the key molecular components of the egg strings of the salmon louse (*Lepeophtheirus salmonis*). Genes.

[CR8] Boxaspen K (2006). A review of the biology and genetics of sea lice. ICES J Mar Sci J du Cons.

[CR9] Boxaspen K, Næss T (2000). Development of eggs and the planktonic stages of salmon lice (Lepeophtheirus salmonis) at low temperatures. Contrib to Zool.

[CR10] Bozinovic G, Sit TL, Hinton DE, Oleksiak MF (2011). Gene expression throughout a vertebrate’s embryogenesis. BMC Genomics.

[CR11] Bricknell IR, Dalesman SJ, O’Shea B (2006). Effect of environmental salinity on sea lice *Lepeophtheirus salmonis* settlement success. Dis Aquat Organ.

[CR12] Bui S, Geitung L, Oppedal F, Barrett LT (2020). Salmon lice survive the straight shooter: a commercial scale sea cage trial of laser delousing. Prev Vet Med.

[CR13] Chen R, Swale DR (2018). Inwardly rectifying potassium (Kir) channels represent a critical ion conductance pathway in the nervous systems of insects. Sci Rep.

[CR14] Chen ZF, Tian YS, Ma WH, Zhai JM (2020). Gene expression changes in response to low temperatures in embryos of the kelp grouper.

[CR15] Costello MJ (2009). The global economic cost of sea lice to the salmonid farming industry. J Fish Dis.

[CR16] Crosbie T, Wright D, Oppedal F (2019). Effects of step salinity gradients on salmon lice larvae behaviour and dispersal. Aquac Environ Interact.

[CR17] El Moukhtari A, Cabassa-Hourton C, Farissi M, Savouré A (2020) How does proline treatment promote salt stress tolerance during crop plant development? Front Plant Sci 11:1127. 10.3389/FPLS.2020.01127 10.3389/fpls.2020.01127 PMC7390974 32793273

[CR18] Frantzen M, Bytingsvik J, Tassara L (2020). Effects of the sea lice bath treatment pharmaceuticals hydrogen peroxide, azamethiphos and deltamethrin on egg-carrying shrimp (Pandalus borealis). Mar Environ Res.

[CR19] Gomes SIL, Gonçalves MFM, Bicho RC (2018). High-throughput gene expression in soil invertebrate embryos – mechanisms of Cd toxicity in Enchytraeus crypticus. Chemosphere.

[CR20] Gravil HR (1996) Studies on the biology and ecology of the free swimming larval stages of Lepeophtheirus salmonis (Kröyer, 1838) and Caligus elongatus Nordmann, 1832 (Copepoda: Caligidae). University of Stirling

[CR21] Groner M, Laurin E, Stormoen M (2019). Evaluating the potential for sea lice to evolve freshwater tolerance as a consequence of freshwater treatments in salmon aquaculture. Aquac Environ Interact.

[CR22] Hahnenkamp L, Fyhn HJ (1985). The osmotic response of salmon louse,Lepeophtheirus salmonis (Copepoda: Caligidae), during the transition from sea water to fresh water. J Comp Physiol B.

[CR23] Hammond LTM, Hofmann GE (2012). Early developmental gene regulation in Strongylocentrotus purpuratus embryos in response to elevated CO2 seawater conditions. J Exp Biol.

[CR24] Hamre L, Bui S, Oppedal F (2019). Development of the salmon louse *Lepeophtheirus salmonis* parasitic stages in temperatures ranging from 3 to 24°C. Aquac Environ Interact.

[CR25] Hamre LA, Glover KA, Nilsen F (2009). Establishment and characterisation of salmon louse (*Lepeophtheirus salmonis* (Krøyer 1837)) laboratory strains. Parasitol Int.

[CR26] Hansen BW (2019). Copepod embryonic dormancy: “An egg is not just an egg.”. Biol Bull.

[CR27] Harðardóttir HM, Male R, Nilsen F, Dalvin S (2019). Effects of chitin synthesis inhibitor treatment on Lepeophtheirus salmonis (Copepoda, Caligidae) larvae. PLoS One.

[CR28] Helgesen KO, Horsberg TE, Tarpai A (2019). The surveillance programme for resistance to chemotherapeutants in salmon lice (Lepeophtheirus salmonis) in Norway 2018.

[CR29] Hjeltnes B, Bang-Jensen B, Bornø G (2018). The health situation in Norwegian aquaculture 2017.

[CR30] Holm MW, Kiørboe T, Brun P (2018). Resting eggs in free living marine and estuarine copepods. J Plankton Res.

[CR31] Jensen EM, Horsberg TE, Sevatdal S, Helgesen KO (2020). Trends in de-lousing of Norwegian farmed salmon from 2000–2019—consumption of medicines, salmon louse resistance and non-medicinal control methods. PLoS One.

[CR32] Johnson SC, Albright LJ (1991). Development, growth, and survival of lepeophtheirus salmonis (Copepoda: Caligidae) under laboratory conditions. J Mar Biol Assoc United Kingdom.

[CR33] Karlsson K, Puiac S, Winder M (2018). Life-history responses to changing temperature and salinity of the Baltic Sea copepod *Eurytemora affinis*. Mar Biol.

[CR34] Kassambara A (2020) ggpubr: “ggplot2” Based Publication Ready Plots. https://cran.r-project.org/package=ggpubr

[CR35] Kind KL, Collett RA, Harvey AJ, Thompson JG (2005). Oxygen-regulated expression of GLUT-1, GLUT-3, and VEGF in the mouse blastocyst. Mol Reprod Dev.

[CR36] Lee J, Phillips MC, Lobo M, Willett CS (2021). Tolerance patterns and transcriptomic response to extreme and fluctuating salinities across populations of the intertidal copepod Tigriopus californicus. Physiol Biochem Zool.

[CR37] Moltumyr L, Gismervik K, Gu J (2021). Does the thermal component of warm water treatment inflict acute lesions on Atlantic salmon (Salmo salar)?. Aquaculture.

[CR38] Montory JA, Cumillaf JP, Cubillos VM (2018). Early development of the ectoparasite Caligus rogercresseyi under combined salinity and temperature gradients. Aquaculture.

[CR39] Powell MD, Reynolds P, Kristensen T (2015). Freshwater treatment of amoebic gill disease and sea-lice in seawater salmon production: considerations of water chemistry and fish welfare in Norway. Aquaculture.

[CR40] R Core Team (2020) R: a language and environment for statistical computing. https://www.r-project.org/

[CR41] Runcie DE, Garfield DA, Babbitt CC (2012). Genetics of gene expression responses to temperature stress in a sea urchin gene network. Mol Ecol.

[CR42] Sievers M, Oppedal F, Ditria E, Wright DW (2019). The effectiveness of hyposaline treatments against host-attached salmon lice. Sci Rep.

[CR43] Skern-Mauritzen R, Malde K, Eichner C (2021). The salmon louse genome: copepod features and parasitic adaptations. Genomics.

[CR44] Stone J, Boyd S, Sommerville C, Rae GH (2002). An evaluation of freshwater bath treatments for the control of sea lice, Lepeophtheirus salmonis (Kroyer), infections in Atlantic salmon, Salmo salar L. J Fish Dis.

[CR45] Sutherland BJG, Jantzen SG, Yasuike M (2012). Transcriptomics of coping strategies in free-swimming *Lepeophtheirus salmonis* (Copepoda) larvae responding to abiotic stress. Mol Ecol.

[CR46] Thompson J, Lane M, Robertson S (2006) Adaptive responses of early embryos to their microenvironment and consequences for post-implantation development. Adv Exp Med Biol 573:58–69

[CR47] Tomancak P, Beaton A, Weiszmann R (2002). Systematic determination of patterns of gene expression during Drosophila embryogenesis. Genome Biol.

[CR48] Vollset KW, Dohoo I, Karlsen Ø (2018). Disentangling the role of sea lice on the marine survival of Atlantic salmon. ICES J Mar Sci.

[CR49] Voolstra CR, Schnetzer J, Peshkin L (2009). Effects of temperature on gene expression in embryos of the coral Montastraea faveolata. BMC Genomics.

[CR50] Wright DW, Geitung L, Karlsbakk E (2018). Surface environment modification in Atlantic salmon sea-cages: effects on amoebic gill disease, salmon lice, growth and welfare. Aquac Environ Interact.

[CR51] Wright DW, Oppedal F, Dempster T (2016). Early-stage sea lice recruits on Atlantic salmon are freshwater sensitive. J Fish Dis.

